# Diabetic Kidney and Liver Disease Concepts

**DOI:** 10.1111/1753-0407.70163

**Published:** 2025-10-30

**Authors:** Zachary T. Bloomgarden

**Affiliations:** ^1^ Department of Medicine, Division of Endocrinology, Diabetes and Bone Disease Icahn School of Medicine at Mount Sinai New York New York USA

**Summary**

- This commentary on recent publications in the field summarizes research findings on diabetic ketoacidosis (DKA) in people with type 2 diabetes (T2D) treated with sodium‐glucose cotransporter 2 inhibitors (SGLT2i), on the renal and cardiovascular benefits of mineralocorticoid receptor antagonists (MRA), on glucagon‐like peptide‐1 receptor agonists (GLP‐1RA), and on new approaches to metabolic dysfunction‐associated steatohepatitis (MASH).
- *DKA characteristics in SGLT2i users*: Analysis of 1060 DKA cases in T2D patients showed that those treated with SGLT2i had slightly lower blood glucose but greater acidosis compared to nonusers, with similar hospital stay duration and ICU admissions. Suboptimal diabetes management was noted in SGLT2i users developing DKA, highlighting prevention needs.
- *Renal effects of empagliflozin*: In 23 340 participants from four trials, empagliflozin showed renal benefits regardless of diabetes status or albuminuria level. Acute eGFR dips were common but not linked to adverse outcomes, and empagliflozin reduced risks of kidney injury, eGFR decline, and kidney failure, with greater benefits in diabetic patients but also in those with lower levels of albuminuria. Early initiation is favored.
- *Additive benefits of finerenone and empagliflozin*: In 800 patients with albuminuria, combining finerenone and empagliflozin reduced albuminuria by 29% more than finerenone alone and 32% more than empagliflozin alone, with similar effects regardless of GLP‐1RA use, suggesting complementary cardiorenal benefits among these drug classes.
- *Semaglutide and MRA combination effects*: Among 3533 T2D patients with CKD, semaglutide reduced renal events by 49% in those on MRA and by 21% in those not on MRA, with comparable eGFR preservation, indicating potential additive renal protection from combined therapy.

## 1 Renal Disease, SGLT2i, MRA, and GLP‐1RA

Using individual‐level data from 23 340 participants in four placebo‐controlled trials, EMPA‐REG OUTCOME (7020 people with T2D and previous ASCVD), EMPEROR‐Reduced and EMPEROR‐Preserved (9711 with heart failure), and EMPA‐KIDNEY (6609 with CKD at risk of progression), the authors studied the benefit of the sodium‐glucose countertransporter 2 inhibitor (SGLT2i) empagliflozin when albuminuria is low, in the absence of diabetes, and among those with large acute estimated glomerular filtration rate (eGFR) dips on initiation of treatment, calculating off‐treatment dip‐free eGFR slopes by dividing the difference between off‐treatment eGFR and randomization eGFR by the time between the two measurements in the 10 630 (45.5%) participants with measurement of off‐treatment eGFR after discontinuing study treatment, of whom 6250 were allocated to empagliflozin [1]. The independent predictors of larger acute eGFR dips included older age (0.9% larger dip per 10 years), higher eGFR (4.6% larger dip for eGFR ≥ 85 vs. < 37 mL/min per 1.73 m^2^) and level of albuminuria (2.9% larger dip for urine albumin/creatinine ratio [UACR] ≥ 324 vs. < 6 mg/g), renin–angiotensin system inhibitor and diuretic use (1.1% and 0.8% larger dips with use vs. nonuse, respectively), higher systolic blood pressure (0.9% larger dip per 17 mmHg), lower hematocrit (0.9% larger dip per 4.8%), and lower BMI (0.5% larger dip per 5.8 kg/m^2^). A 4‐week eGFR dip of more than 14% was seen in 2948 (24.5%) of 12 031 participants in the empagliflozin groups and 1219 (12.5%) of 9761 participants in the placebo groups, with 311 (2.6%) of those randomized to empagliflozin versus 154 (1.6%) of participants receiving placebo having an acute eGFR dip of more than 30%. The likelihood of a 50% or greater increase in follow‐up serum creatinine among those allocated to empagliflozin was reduced by 20%, regardless of predicted size of acute eGFR dip, diabetes status, baseline eGFR, or albuminuria, previous heart failure, or by baseline NT‐proBNP. Similarly, allocation to empagliflozin reduced the risk of acute kidney injury by 27% regardless of predicted size of acute eGFR dip, and this was again consistent in the other subgroups. The likelihood of a 40% or greater decrease in eGFR from baseline was reduced by 30%, and the likelihood of development of kidney failure was reduced by 34% with empagliflozin. The annual rate of decline in chronic eGFR slope decreased by 64% with empagliflozin, and this was greater among participants with diabetes (74%) than those without diabetes (42%). The relative decrease in chronic eGFR slope was 81%, 69%, 59%, and 41% less in participants with UACR < 30, 30 to < 300, 300 to < 1000, and 1000 or more, respectively, which the authors consider an argument in favor of starting SGLT2i early rather than waiting for albuminuria to develop. The authors suggest that, although acute increase in creatinine is listed as an adverse effect in the product label for empagliflozin and other SGLT2i, the analysis suggests this is not associated with adverse outcome, and they “discourage routine remeasurement of eGFR after initiation.”

In a study of 800 patients with micro‐ or macroalbuminuria, the additive benefit of the combination of finerenone and empagliflozin in reducing albuminuria was 29% greater than that of finerenone alone and was 32% greater than that of empagliflozin alone [2]. The effect was similar in those receiving or not receiving a GLP‐1RA at baseline, implying these three classes, along with the use of agents affecting the renin‐angiotensin‐aldosterone system, likely can all be considered to be complementary in their cardiorenal benefits [3].

A similar analysis compared the effect of randomization of 3533 patients with T2D and CKD to a 3.4‐year period of semaglutide versus placebo among the 257 receiving a mineralocorticoid receptor antagonist (MRA) at baseline versus those not receiving this treatment [4]. In the group receiving a MRA, a ≥ 50% reduction in eGFR from baseline or other renal outcome occurred in 17% with semaglutide but in 30% among those not randomized to semaglutide, a 49% reduction in renal events, while in those not receiving a MRA a renal outcome occurred in 19% with semaglutide but in 23% among those not randomized to semaglutide, a 21% reduction. Although the test for interaction showed this not to be statistically significant, the numeric difference certainly appears to favor the use of both semaglutide and a MRA. The mean reduction in eGFR, based either on creatinine or on cystatin C, showed similar protection with semaglutide among those receiving versus not receiving a MRA.

Analysis of 1060 diabetic ketoacidosis (DKA) cases in people with T2D compared the 267 cases in SGLT2i‐treated persons with cases in 793 nonusers [5]. Median diabetes duration was 10 years, SGLT2i use 2 months; admission HbA1c was 9.2%, ketones 5.7, lactate 1.5, and bicarbonate 8.8 mmol/L, pH 7.1, glucose 191 and creatinine 1.1 mg/dL; mean DKA duration was 48 h. In a precipitating cause‐matched comparison of SGLT2i users versus nonusers, glucose was 360 versus 463, pH 7.1 versus 7.2, lactate 2.7 versus 3.5, and ketones 5.5 versus 5.4 mmol/L. Hypokalemia occurred in 49% versus 39% of SGLT2i users versus nonusers, and length of hospital stay and ICU admissions were similar. On balance, although SGLT2i users had somewhat lower blood glucose levels and greater degrees of acidosis, the differences were modest. Of greatest note is the suboptimal diabetes management in SGLT2i users developing DKA, suggesting a reasonable approach for prevention.

## 2 Metabolic Dysfunction‐Associated Steatohepatitis (MASH)

Fibroblast growth factor 21 (FGF21) is synthesized mainly in the liver and enhances hepatic insulin sensitivity, reduces hepatic triglyceride accumulation, having anti‐inflammatory, antioxidant, and antifibrotic effects, with several FGF21 analogs in development [6]. In a Phase 2 Study of 126 people with biopsy‐proven steatohepatitis and histological stage F2 or F3 fibrosis treated with the FGF21 analog efruxifermin versus placebo for 96 weeks, ≥ 1‐stage fibrosis improvement without MASH worsening occurred in eight (24%) of 34 participants in the placebo group, 12 (46%) of 26 in the 28 mg group, and 21 of 28 in the 50 mg group [7]. Liver fat decreased 10% with placebo, and 34% and 41% with efruxifermin 28 and 59 mg, and adiponectin increased 17%, 28%, and 63%, respectively, with evidence of reduction in insulin resistance, improvement in lipids, decrease in alanine aminotransferase and aspartate aminotransferase, and decreased liver stiffness on transient elastography imaging. Eight of 27 participants treated with 50 mg efruxifermin showed near‐complete reversal of disease. Side effects included diarrhea, nausea, emesis, and increased appetite. Bone mineral density was unchanged at week 48, although decreases were seen at week 96, without increased fractures.

In an individual participant analysis of the relationship between metabolic response to tirzepatide and improvement in liver bx‐confirmed MASH among 154 participants, 59% with T2D, 80 were responders with resolution of MASH and no worsening of fibrosis, of whom 76 received tirzepatide. Responders had 16% weight loss, while non‐responders had 7% weight loss [8]. Responders had a greater reduction in HbA1c, triglyceride, and liver fat, and a greater increase in insulin sensitivity and adiponectin. Improvement in fibrosis was similarly associated with greater weight loss and improvement in HbA1c. Weight loss correlated with liver fat, liver stiffness, and inflammation.

Oligodendrocytes (OL) are specialized cells in the CNS which act along with astrocytes and microglia in supporting neuronal function, the OL producing myelin sheaths that comprise a large fraction of brain white matter, with involvement in learning and memory and in neuronal circuit remodeling; OL are highly dependent on glycolysis for metabolic substrate and in turn provide energy for neuronal axon metabolism [9]. In a study of the role of OL glucose‐dependent insulinotropic polypeptide receptor (GIPR) signaling, Hansford et al. found that the GIPR is highly expressed on OLs, particularly in the median eminence (ME) of the mediobasal hypothalamus, the brain region lacking a blood–brain barrier and hence directly exposed to circulating hormones and nutrients, with peripheral administration of a labeled GIP receptor agonist leading to ME OL uptake, while OL‐specific GIPR deletion led to reduction in ME OL density in association with reduced ME myelin expression and was associated with decreased insulin sensitivity and decreased body weight in a diet‐induced obesity model with loss of the anti‐obesity efficacy of combined GLP‐1/GIP receptor signaling, with evidence that GIP receptor activation increases access of a GLP‐1 agonist to the hypothalamus, suggesting a mechanism by which GIPR agonism can enhance the weight‐loss efficacy of GLP‐1R agonists [10].

## Conflicts of Interest

The author declares no conflicts of interest.
